# End-stage liver disease in alcohol-dependent patients: Case report and overview on clinical management

**DOI:** 10.1007/s40211-019-0314-5

**Published:** 2019-06-19

**Authors:** Stephan Listabarth, Daniel König, Andrea Gmeiner, Andreas Wippel, Benjamin Vyssoki

**Affiliations:** Clinical Division of Social Psychiatry, Department of Psychiatry and Psychotherapy, Medical University of Vienna, Währinger Gürtel 18–20, 1090 Vienna, Austria

**Keywords:** Addiction, Liver cirrhosis, Withdrawal, Psychiatry, Interdisciplinary management, Substanzabhängigkeit, Leberzirrhose, Entzug, Psychiatry, Interdisziplinäre Behandlung

## Abstract

This case report is about a 44-year-old woman with alcohol-related end-stage liver disease. Initial contact with the patient was made in the alcohol-outpatient clinic of the Department of Psychiatry and Psychotherapy, Clinical Division of Social Psychiatry, Medical University of Vienna. Due to a particularly poor general condition, Child Pugh Score C/MELD Score 20, the patient was admitted to ward 4A, with the clinical and scientific focus of treating patients with alcohol use disorder. The withdrawal process was complicated by a multitude of factors associated with end-stage liver disease. By explaining the theoretical background of possible somatic as well as psychiatric complications of end-stage liver disease and elaborating on treatment options a comprehensive overview of the psychiatric and somatic management of this patient population is given.

## Zusammenfassung

Im vorliegenden Beitrag wird der Fall einer 44-jährigen Frau mit alkoholassoziierter Lebererkrankung im Endstadium beschrieben. Die Erstvorstellung der Patientin erfolgte in der Ambulanz für Alkoholismusgefährdete der Klinischen Abteilung für Sozialpsychiatrie, Universitätsklinik für Psychiatrie und Psychotherapie, Medizinische Universität Wien. Aufgrund eines ausgesprochen schlechten Allgemeinzustands mit Child-Pugh-Score C und MELD-Score 20 wurde die Patientin auf der Station 4A, mit klinischem und wissenschaftlichem Schwerpunkt der Behandlung von Patienten mit Alkoholabhängigkeit, aufgenommen. Der medikamentös-gestützte Entzug war durch eine Vielzahl an Faktoren, die mit einer Lebererkrankung im Endstadium zusammenhingen, erschwert. Im Beitrag wird der theoretische Hintergrund möglicher somatischer wie auch psychiatrischer Komplikationen der Lebererkrankung im Endstadium erläutert, zudem werden die Behandlungsoptionen ausführlich beschrieben. Es wird ein umfassender Überblick des psychiatrischen und somatischen Managements dieser Patientengruppe geboten.

## Introduction

Alcohol use disorder (AUD) and its associated secondary diseases are a significant burden, from an individual as well as from a public health perspective, worldwide, but especially in Europe. Globally, 3.3 million deaths per year (5.9% of all deaths) and 5.1% of all disability-adjusted life years (DALYs) are postulated to be the immediate result of alcohol consumption [1]. Among the conditions and diseases caused by increased alcohol consumption, end-stage liver disease (i.e. liver cirrhosis) is the most considerable one, as in 2010, 1.8% of all deaths in Central Europe were due to alcohol-induced liver cirrhosis (AIL) [2]. Life expectancy for patients suffering from AIL is significantly reduced, with 3‑month mortality rates ranging from 6.0% up to 71.3%. The only curative treatment, due to the irreversible damage inflected on the liver tissue, is liver transplantation. Besides the complexity of the surgical procedure and the potential complications, a major barrier of liver transplantation for most patients is the so-called “6-month rule”, which requires patients to be abstinent for at least 6 months before even being listed for transplantation. Concurrent with a typically low therapy adherence within this patient collective, the 6‑month rule represents a significant burden for patients as well as care-givers. Clinical signs and symptoms of this disease include rather harmless ones, like spider angioma, palmar erythema, jaundice, angular cheilitis, and a coated tongue, but also major complications like ascites, coagulopathy and hepatic encephalopathy and even potentially lethal conditions like spontaneous bacterial peritonitis (SBP) and hepatocellular carcinoma. In addition to these somatic conditions, treatment options in the psychiatric management of these patients are limited, due to altered pharmacokinetics caused by liver damage.

## The case

A 44-year-old woman with a history of polysubstance abuse, in particular opioid and alcohol dependence, was admitted for in-patient care, after consulting the outpatient clinic, due to poor general condition. The patient had previously been admitted to in-patient psychiatric care at the age of 19 due to deliberate self-harm; an emotional-instable personality disorder was diagnosed. The patient had been in a therapeutic opioid substitution program since the age of 25 and reported to suffer from alcohol use disorder since the age of 26. Daily alcohol consumption was at times as high as 384 g of pure alcohol, while the longest period of abstinence was reported with a duration of 6 months. She had not been in psychiatric or psychotherapeutic treatment recently—neither in the in-patient nor out-patient setting. In her somatic history, the patient had previously been diagnosed with alcohol-induced liver cirrhosis, pulmonal hypertension, splenomegaly and had already undergone three instances of band ligation due to hemorrhaging of esophageal varices.

Initially, the patient had been seeking medical attention due to progressive edemas of the lower extremities with stasis eczema. Physical examination and initial diagnostics revealed additional signs of decompensated liver cirrhosis: reduced general condition, ascites, enlarged liver and splenomegaly, severe pancytopenia (leukocytes 2.65 G/L, thrombocytes 30 G/L), elevated serum ammonia levels, jaundice with elevated bilirubin levels and liver enzymes (De Ritis ratio 1.96). These findings result in a Child Pugh Score of 12, equivalent with Stadium C, and a MELD score of 20 points, indicating a 3 month mortality of 19.6%. At the time of hospitalization, the diuretic therapy with furosemide was changed to spironolactone—which should be given preference in patients with liver disease. When blood tests revealed the patient to be suffering from hyperammonemia, Spironolactone was paused and to address elevated ammonia levels, L‑ornithine-L-aspartate was administered intravenously and lactulose orally. Furthermore, for hypoalbuminemia, the patients received human albumin intravenously. Additionally, a prophylactic therapy against recurrence of hyperammonemia and possible hepatic encephalopathy with the antibiotic rifaximine was established. Also, thiamine was given intravenous for 5 days in a dosage of 300 mg per day. Thereafter, thiamine substitution was continued perorally. Vitamin K was parenterally administrated for the first three days of the in-patient stay, on the one hand, to test the remaining synthesis capacity of the liver (see Koller Test in the discussion) and, on the other hand, to improve coagulation by an increased internationalized ratio (INR).

By reason of a positive medical history for multiple instances of hemorrhaging of esophageal varices, antihypertensive therapy with carvedilol was established to minimize the risk for this complication. Although bilirubin levels kept rising in the early stages of the in-patient stay and jaundice was becoming more pronounced, the decision of a watchful-waiting procedure was made, and with the improvement of the general condition of the patient, the bilirubin level eventually decreased to values within the reference range. Due to symptoms of a urinary tract infection, an antibiosis with nitrofurantoin was started. Whereas dysuria and urine tests improved with this therapy, simultaneously the previously documented thrombocytopenia aggravated, and intravenous substitution of thrombocytes was evaluated. With thrombocytes at 22 G/L, antibiosis was stopped after seven days. However, no recurrence of any signs of urinary tract infections occurred during the further stay and thrombocyte levels stabilized within the next weeks, with undulating values around 30 G/L.

As abdominal ultrasound had shown an unspecific mass in liver segment III, an additional magnetic resonance cholangiopancreaticography (MRCP) was conducted, in which a hypervascularized lesion was confirmed and consistent follow-ups were recommended. A cerebral magnetic resonance imaging (MRI) revealed signs of chronic neurodegenerative processes within the context of liver cirrhosis, in particular in the area of globus pallidus, hypothalamus and adenohypophysis and, moreover, multiple areas of microbleedings and a global reduction of brain substance. On the fourth day of the in-patient stay a gastroscopy was conducted and revealed a severe hypertensive gastropathy (a typical consequence of portal hypertension)—presence of current varices was, however, excluded.

Due to decreasing ammonia levels and the absence of clinical signs or symptoms of hepatic encephalopathy, therapy with lactulose could be halted during the further stay. It was also possible to re-establish the diuretic therapy with spironolactone, which lead to significant weight reduction and further improvement of the peripheral edema and severity of ascites. In the further course magnesia and folic acid were substituted, due to deficiency displayed in constant laboratory examinations.

Hereafter psychiatric management of the patient will be discussed. Due to the favorable pharmacokinetics of this specific benzodiazepine in presence of liver damage, oxazepam was used for alcohol withdrawal—the initial daily dosage of 200 mg was reduced stepwise, until it could be stopped without occurrence of withdrawal complications. Due to the previously documented liver damage, baclofen was established in the further course of the stay as anticraving therapy and relapse prevention, instead of the often used naltrexone or acamprosate. The existing substitution with morphine hydrochloride (Compensan) was continued during the whole stay. During the stay, the patient presented with persistent symptoms of depression after withdrawal and antidepressive therapy with milnacipran was begun. The initial daily dosage of 25 mg was stepwise increased to an eventual daily dosage of 100 mg, which yielded a sufficient response. As the patient reported sleeping disorders, soporific therapy with 25 mg quetiapine was established.

After more than four weeks the patient was stable and transferred to an institution specialized in long-term rehabilitation and weaning.

## Discussion

The primary goals in the clinical management of patients with AIL are stabilization of the general condition and prevention of life-threatening complications.

As the first diagnostic tool, extensive laboratory tests are recommended. Common findings within this group of patients are anemia and other blood count abnormalities, like thrombocytopenia and leukopenia. Furthermore, electrolyte imbalances, elevated liver enzymes, bilirubin as well as ammonia are typical findings. Two mechanisms causing anemia in AUD patients are discussed: First, it is supposed to be a result of alcohol-induced lack of vitamin B12 and folic acid and second, by a direct myelodepressive effect of alcohol itself [3]. The latter mechanism is also an explanation why thrombocytes and leukocytes can also be affected, eventually resulting in pancytopenia. Especially, leukocytopenia can have crucial implications, like an elevated susceptibility for infections. Furthermore, in patients with AIL, the immune system is additionally impaired due to a reduced liver synthesis function. Both, anemia and leukopenia, are usually self-limiting without any specific treatment necessary, as long as the patient remains abstinent from alcohol. An exception in this respect is the obligatory substitution of folic acid, vitamin B12 and iron, if indicated by laboratory tests. Concerning the iron metabolism, it is noteworthy that the majority of AUD patients do not have a lack of iron, but rather show highly elevated levels of ferritin and an abnormal high transferrin saturation. However, these observed effects of the iron homeostasis are reversible within a few days of abstinence.

Elevated liver enzymes are an important diagnostic factor in patients with AUD. The so-called “De Ritis ratio” is calculated of the relation between aspartate transaminase (AST) and alanine transaminase (ALT) serum levels and provides an indication of the pathogenesis of liver damage and is helpful for differential diagnostic considerations. If the AST-ALT ratio is smaller than 1, this is a strong indicator for a liver disease caused by a viral infection [4], whereas values between 1 and two indicate an increased alcohol consumption and a ratio higher than 2 is typical for an acute alcohol induced hepatitis.

Another relevant parameter for the efficiency of the liver function is the INR, which is often prolonged in patients with AIL. The INR mainly depends on the activity of coagulation factors II, V, VIII and X—all of them synthesized in the liver. By applying vitamin K intravenous and re-evaluating the coagulation test (INR) after at least 24 h, this circumstance can also be used for diagnostic measures. Improving INR following vitamin K substitution is indicative of residual liver function, whereas an absent improvement can be seen as a sign of more severe liver damage [5]. Further aspects of liver synthesis disorders are reduced albumin levels and alteration of the protein profile (i.e. a decrease in the albumin–globulin ratio). Clinically, these changes manifest themselves in osmotic leaking of fluid into the tissue, resulting in ascites, peripheral edema or even pericardial effusion. Therefore, patients with decompensated AIL are often treated with human albumin solution.

Moreover, hepatic encephalopathy (HE) is a potentially serious complication in patients with AIL. Diagnostic measurements consist of monitoring ammonia serum level and clinical evaluation of the patient. It should be noted that the presented signs and symptoms can differ significantly depending on the severity of the condition, and especially mild forms of HE might be overseen in some cases [6]. These so-called covert HE are mild, but still associated with frequent falls, fatigue, disinterest and distraction [6]. Clinical symptoms range from mildly reduced consciousness and attention to disorientation, confusional states and in the most extreme case even to comatose states. Although the exact pathophysiology of HE is controversially discussed, a significant role of ammonia is commonly postulated. Increased serum levels of ammonia impose a neurotoxic effect and inhibit excitatory neurotransmission [6]. In case of an abrupt rise of ammonia levels, as can be observed in acute alcoholic hepatitis, the emergence of a brain edema is a possible and harmful complication. West Haven and FOUR criteria are scores that can be used to objectively grade the severity of HE in regard to clinical symptoms.

All the above-mentioned aspects and complications respectively, can also be used to differ between the different stages of alcohol-induced liver diseases: Scores, like the Child–Pugh Score or the Model of End-Stage Liver Disease (MELD) Score, initially established to evaluate the urgency for transplantation, are also important in estimating the prognosis of this patients. The MELD Score is calculated by creatinine and bilirubin serum levels and the INR, whereas the Child–Pugh Score additionally takes the presence of ascites or HE into account. The prognostic outcome stated by these scores is striking: Two-year mortality of patients with a Child–Pugh Score equaling class C is as high as 75%, and the three-month mortality in patients with a MELD Score of 30 to 39 points is around 50%. If the MELD Score is higher than 40 points the three-month mortality exceeds 70%.

A relatively new diagnostic tool in the evaluation of the liver tissue is the transient elastography (FibroScan®, Echosens, Paris, France). This method allows noninvasive examination of the liver parenchyma by combining an ultrasound probe with an additional transducer, transmitting pressure waves, the expansion of which is then analyzed. In this way, the degree of elasticity can be measured, which represents an indirect parameter for the degree of cirrhotic transformation: The higher the measured values (kilopascal, kPa), the stiffer the liver tissue is. In AIL the stiffness is due to progressing incorporation of connecting tissue fibers, i.e. fibrosis, into the liver parenchyma. The measured elasticity allows the examiner to differentiate between the specific stages of fibrosis (F0–F4), although there are also other reasons for high values other than AIL, for example pre-examination nourishment, systemic diseases affecting the liver or viral infections.

As far as the alcohol withdrawal is concerned, a number of intricacies need to be considered in patients with AIL. The recommended substance for pharmacological-supported withdrawal in this group of patients is oxazepam, due to several advantages in pharmacokinetics compared to other benzodiazepines. First, oxazepam is solely glucuronized in the liver, and not metabolized by the cytochrome P450 enzymes (CYPs), which are often reduced in their functionality in case of liver disease [7]. Second, oxazepam has a relatively short half-life and therefore allows accessible and immediate dose adjustments. Third, there are no additional active metabolites emerging during the metabolization of oxazepam, making it, again, more manageable. Lorazepam, which can be used as an alternative, is also metabolized via glucuronidation, but features a slightly longer half-life than oxazepam (10–20 h for lorazepam compared to 4–15 h for oxazepam).

Another important aspect in the management of AUD patients with end-stage liver disease is the pharmacological support in reducing craving and preventing relapses. The only three substances available on the market in Europe, naltrexone, nalmefene and acamprosate, are not approved for patients with end-stage liver disease. Therefore, there are no regularly approved drugs for these patients, which is notable, since the common presence of AUD and end-stage liver diseases is very likely, due to the causal relation. There have been efforts to examine alternative substances for this particular collective of patients: One randomized, double-blinded study, for example, indicates a significant improvement of abstinence rates for patients treated with baclofen [8]. In this study, the initial dosage of baclofen was three times 5 mg per day, increased to a recommended daily dosage of 30 mg during further course. However, the existing evidence for the efficacy of baclofen is limited and a recent systematic review concludes that baclofen is not superior to placebo in regard to relapse rate and craving symptoms [9]. Further research in this area is needed, to facilitate an optimal evidence-based therapy approach for a group of patients, in whom relapses can imply severe consequences.

The treatment of psychiatric comorbidities, like depression or anxiety, in patients suffering from AUD can be another psychopharmacological challenge. Here again, the altered pharmacokinetics, due to a reduced liver metabolism, should be considered carefully. The only exception here is milnacipran, which only gets glucuronized within the liver and is then eliminated renally. All other agents of selective serotonin reuptake inhibitors (SSRIs) or serotonin and noradrenalin reuptake inhibitors (SNRIs) should only be prescribed with adapted dosages. In Table 1 recommendations for antidepressant therapy of patients with AUD are summarized [10].

**Table 1:** Antidepressants in end-stage liver disease

No Adaption of Dosage Necessary	(Levo)Milnacipran
No Adaption in Case of Minor to Mild Liver Dysfunction Necessary	Escitalopram Vortioxetine
Restricted Use Possible	Bupropion Citalopram Venlafaxine Fluoxetine Mirtazapine Paroxetine Amitriptyline
Avoid	Duloxetine
No Sufficient Data Available	Sertraline

## Conclusion

With the aid of this case we describe the challenges and major clinical aspects in the treatment of AUD patients with end-stage liver disease. Complemented by theoretical background, the necessary diagnostic and therapeutic measurements are explained. All in all, it is important to be cautious of potentially lethal complications and keep possible hepatotoxic effects or modified pharmacokinetics for this group of patients in mind. Medical anticraving and relapse-prevention is from uttermost importance for these patients, especially when considering the 6‑month rule that needs to be achieved before being listed for a new liver. There are a few approved pharmacological options in supporting patients to reach this goal; however, further research in this area is needed. Besides acute inpatient management, it is also important to support the patient to ensure continuous and comprehensive aftercare, including psychotherapy and psychosocial support and interventions.
